# CD200在鉴别诊断经典型毛细胞白血病与变异型毛细胞白血病中的价值

**DOI:** 10.3760/cma.j.issn.0253-2727.2023.09.013

**Published:** 2023-09

**Authors:** 静 余, 四书 赵, 肖 陈, 纯 乔, 蓉 王, 建勇 李, 雨洁 吴

**Affiliations:** 南京医科大学附属第一医院（江苏省人民医院）血液科，南京 210029 Department of Hematology, the First Affiliated Hospital of Nanjing Medical University, Jiangsu Province Hospital, Nanjing 210029, China

毛细胞白血病（HCL）和变异型毛细胞白血病（HCL-v）均为罕见的成熟小B细胞淋巴瘤[1]，两者的细胞形态学非常相似，均可表现为毛细胞样，但两者的生物学特征及治疗方案完全不同[2]–[3]。因此，精准诊断HCL和HCL-v对于临床非常重要。多参数流式细胞术（MFC）是诊断和鉴别诊断HCL和HCL-v的重要检测方法，目前主要利用CD25和CD123两个标志进行区分，大多数HCL中CD25和CD123为阳性，而大多数HCL-v中CD25和CD123为阴性[4]–[5]。部分患者免疫表型不典型，难以通过CD123和CD25的表达特点进行鉴别。

CD200（OX-2）是一种免疫球蛋白超家族膜糖蛋白，在B细胞、胸腺细胞、活化的T细胞、神经元和内皮细胞树突细胞等多种细胞中均表达，主要通过与其受体（CD200R）的相互作用发挥免疫抑制作用[6]。目前国内外文献主要集中于CD200在慢性淋巴细胞白血病（CLL）与套细胞淋巴瘤（MCL）鉴别诊断中的价值[7]，CD200在HCL和HCL-v中诊断价值的国内相关研究较少。本研究回顾性分析了CD200在HCL和HCL-v中的免疫表型特点，结合免疫组化和分子生物学特征，探索CD200在HCL和HCL-v中的表达特点和诊断价值。

## 病例与方法

1. 病例：回顾性分析2016年1月至2022年5月南京医科大学第一附属医院（江苏省人民医院）收治的初诊HCL和HCL-v患者19例，诊断参照2016年WHO淋巴系统肿瘤分类[8]。所有患者均结合临床生物学特点、形态学、免疫表型、细胞遗传学、BRAF V600E表达、免疫球蛋白重链可变区（IGHV）基因突变情况进行综合分析。本研究得到南京医科大学第一附属医院（江苏省人民医院）伦理委员会的批准，所有患者均签署知情同意书。

2. 主要仪器及试剂：流式细胞仪机型为Beckman Navios（美国Beckman公司），数据分析采用Kaluza流式细胞分析软件（美国）。荧光标记鼠抗人单克隆抗体CD5-ECD、CD79b-PE-Cy5.5、CD20-PE-Cy7、CD19-PE-Cy7、CD200-APC、CD45-KO、FMC7-FITC、CD10-PE、CD25-PE-Cy5.5、CD11c-PE-Cy7、CD103-APC、CD22-PB、CD20-PE-Cy7、CD200-PE、CD19-PE-Alexa Fluor 750（美国Beckman公司），KappaFITC、LambdaPE（丹麦Dako公司），荧光显微镜为ZEISS Imager.Z2（德国ZEISS公司）。

3. Flow-CheckPro质控：每次开机进行一次Flow-checkPro的质控，记录前向散射光（FS）和各荧光信号的半峰变异系数（HPCV）值，追踪一定时间内HPCV的变化，了解仪器的运行情况。首先从冰箱取出Flow-Check Pro荧光微球（PN.A 63493）在室温平衡10 min，后将微球颠倒混匀，滴加0.5 ml（15～20滴）到12 mm×75 mm的试管中。执行开机程序，将样品管放至自动上样盘中，选中“Flow check pro”方案上样分析。调整FS电压使3群微球峰置于设定的区域内，调整FL1～FL10的电压，使各荧光直方图中的荧光平均值为5 007。让仪器低速收集5 000 events。检查FS及各荧光参数直方图X轴细胞半高峰变异系数（HPX-CV）值：FL1～FL8的值应在3％以下，FL9～FL10的值应在4％以下，每次测得的X-平均值的差异应在±5％之间，以确保仪器处于稳定状态，并在表格中记录HPCV结果。

4. 流式细胞术检测：无菌操作抽取2 ml左右骨髓液至EDTA抗凝管，用PBS调整细胞数至（5～10）×10^6^/ml，取100 µl，PBS洗涤一次去除游离轻链，加入500 µl红细胞裂解液，10 min后加入等量PBS混匀静置5 min，离心，去上清后加入1 000 µl PBS洗涤一遍，加入抗体。孵育15 min后加入500 µl PBS悬浮细胞上机。每管至少获取50 000个细胞，先以SSC和CD45设门找出淋巴细胞，分析CD19^+^B细胞各抗原表达特征。进一步分析CD19^+^淋巴瘤细胞中CD200的阳性表达率和平均荧光强度（MFI）。CD200阳性判断标准为抗原表达≥20％。

5. 免疫组织化学染色：本研究按照标准方法进行骨髓涂片，骨髓涂片为瑞氏染色。活检使用中性福尔马林固定，使用10％硝酸溶液脱钙，包埋后按标准方法进行HE染色。所有骨髓活检标本行免疫组化染色，使用AQI S300型自动染色（中山市澳泉医疗科技有限公司），3 µm截面。本研究中使用的抗体为Annexin A1（克隆号：EPR19342，中山市澳泉医疗科技有限公司）。

6. 一代测序：提取患者基因组DNA，针对BRAF基因突变热点V600E位点设计引物，由生工生物工程（上海）股份有限公司合成，正义链：5′-TCATAATGCTTGCTCTGATAGGA-3′；反义链：5′-GGCCAAAAATTTAATCAGTGGA-3′。反应条件：95 °C预变性5 min；然后95 °C变性45 s，58 °C退火45 s，72 °C延伸45 s，35个循环；最后72 °C延伸10 min，生工生物工程（上海）股份有限公司测序，测序结果与野生型基因序列比对。

7. 统计学处理：实验数据采用SPSS 26.0进行统计学分析，计数资料用例数表示，计量资料用中位数（范围）表示，计量资料的比较采用*t*检验，计数资料的比较采用Fisher精确检验，相关性分析采用Spearman相关分析。*P*<0.05为差异有统计学意义。

## 结果

1. 基线临床特征：19例患者中，13例为HCL，6例为HCL-v。HCL患者中男11例，女2例，中位年龄56（31～80）岁，7例年龄≤60岁；HCL-v患者中男5例，女1例，中位年龄75.5（48～85）岁，2例年龄≤60岁。HCL患者中9例脾肿大，HCL-v患者中1例脾肿大。HCL患者中，4例WBC升高，9例WBC降低；HCL-v患者中，6例均出现WBC升高。HCL患者中，6例单核细胞百分比降低，2例升高；HCL-v患者中，3例单核细胞百分比降低，1例升高。8例HCL患者进行了IGHV突变检测，7例阳性，1例阴性；6例HCL-v患者进行了IGHV突变检测，4例阳性，2例阴性。

2. CD200表达情况：13例HCL患者CD200表达均为阳性，6例HCL-v患者CD200表达均为阴性（图1、2）。HCL患者CD200的中位MFI为700.77（95％*CI* 500.25～901.29），HCL-v患者CD200的中位MFI为39.32（95％*CI* 11.06～67.57）。HCL和HCL-v组CD200阳性表达率差异有统计学意义（*χ*^2^＝19.000，*P*<0.001），MFI差异有统计学意义（*F*＝23.045，*P*<0.01）。

**图1:**
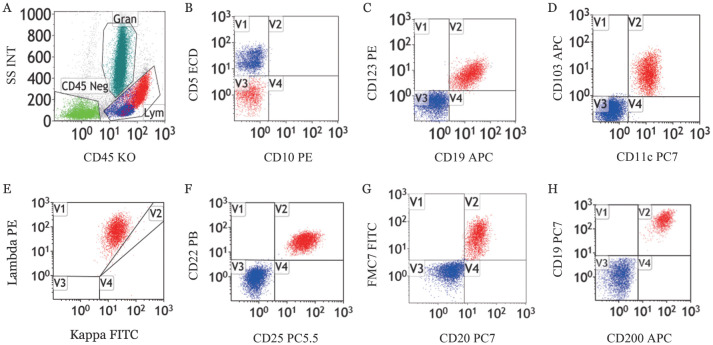
1例毛细胞白血病患者骨髓样本流式细胞术免疫表型 **A** 淋巴细胞（Lym）和粒细胞（Gran）；**B** CD5^−^CD10^−^淋巴细胞；**C** CD19^+^CD123^+^淋巴细胞；**D** CD103^+^CD11c^+^淋巴细胞；**E** CD19^+^Lambda^+^淋巴细胞；**F** CD22^+^CD25^+^淋巴细胞；**G** CD20^+^FMC7^+^淋巴细胞；**H** CD19^+^CD200^+^淋巴细胞，该细胞CD200表达率为98.4％，平均荧光强度为1240

**图2:**
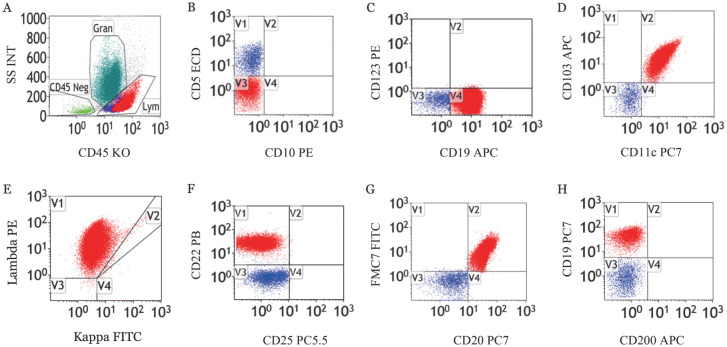
1例变异型毛细胞白血病患者骨髓样本流式细胞术免疫表型 **A** 淋巴细胞（Lym）和粒细胞（Gran）；**B** CD5^−^CD10^−^淋巴细胞；**C** CD19^+^CD123^−^淋巴细胞；**D** CD103^+^CD11c^+^淋巴细胞；**E** CD19^+^Lambda^+^淋巴细胞；**F** CD22^+^CD25^−^淋巴细胞；**G** CD20^+^FMC7^+^淋巴细胞；**H** CD19^+^CD200^−^淋巴细胞，该细胞不表达CD200，平均荧光强度为27

3. BRAF V600E基因突变情况：共11例HCL患者进行了BRAF V600E基因突变检测，其中8例有突变，3例无突变；2例HCL-v患者进行了BRAF V600E基因突变检测，均无突变。

4. Annexin A1检测结果：11例HCL患者骨髓活检免疫组化检测了Annexin A1，9例阳性，2例阴性；6例HCL-v患者均检测了Annexin A1，均为阴性。将CD200与Annexin A1的检测结果进行相关性分析，结果显示，CD200表达与Annexin A1检测结果有中度相关性（*r*＝0.783，*P*<0.01）。

5. CD200表达与CD25、CD123的相关性：13例HCL中，9例患者CD123^+^CD25^+^，2例CD123^−^CD25^−^，1例CD123^+^CD25^−^，1例CD123^−^CD25^+^，但CD123、CD25未同时阳性的4例患者均高表达CD200。6例HCL-v患者中，5例CD123^−^CD25^−^，1例CD123^−^CD25^+^。将CD200、CD25与CD123的检测结果进行相关性分析，CD200表达与CD123（*r*＝0.567，*P*<0.05）和CD25（*r*＝0.716，*P*<0.05）均有中度相关性。

## 讨论

成熟小B细胞淋巴瘤主要包括CLL、MCL、滤泡性淋巴瘤、边缘区淋巴瘤、HCL以及HCL-v等多个亚型，不同亚型的临床、生物学特征及治疗方案也不相同。因此精准诊断对于个体化治疗方案的选择尤为重要[8]–[9]。由于MFC能够通过免疫表型特点快速、客观、准确地检测不同细胞类型及其分化发育阶段，已成为成熟小B细胞淋巴瘤诊断和鉴别诊断中不可或缺的检测技术[10]。但在临床实际工作中，部分病例缺乏典型的免疫表型，导致诊断困难。

HCL是一种罕见的成熟小B细胞淋巴瘤，临床多有脾肿大和全血细胞减少，伴单核细胞减少[1],[11]。HCL-v与典型HCL相似，是一种具有不典型临床特点的脾B细胞淋巴瘤/白血病[12]。与HCL相比，HCL-v通常WBC升高，无单核细胞减少[13]，临床上更具侵袭性，中位生存期短于HCL[14]。形态上，两者的肿瘤细胞均可表现为毛细胞样，因此难以鉴别。在本研究中，13例HCL患者肿瘤细胞均表现为毛细胞样，其中9例患者免疫表型均表现为典型HCL，另有4例HCL患者的CD123和CD25表达不典型，通过Annexin A1与BRAF V600E基因突变检测结果阳性诊断为HCL。6例HCL-v患者的肿瘤细胞均表现为毛细胞样，均出现WBC升高，Annexin A1检测均为阴性，其中5例患者免疫表型为典型HCL-v，另有1例CD123^−^CD25^+^，通过Annexin A1与BRAF V600E基因突变检测结果阴性及其临床表现诊断为HCL-v。

临床通过免疫表型区分HCL和HCL-v，其中80％～90％的典型HCL与HCL-v均不表达CD5和CD10，强表达B细胞标志CD19、CD20、CD22、CD79b和sIg（κ或λ）、CD103和CD11c，两者的主要区别是CD123和CD25，HCL的肿瘤细胞通常表现为CD123^+^CD25^+^，而HCL-v的肿瘤细胞通常表现为CD123^−^CD25^−^[15]–[16]。但在实际工作中发现，部分患者通过CD123和CD25的表达难以明确诊断。本组病例中，有5例HCL或HCL-v患者的CD123和CD25表达不典型，其中免疫表型不典型HCL患者4例，因此探索常规标志以外的标志物表达特点对临床诊断具有重要意义。

CD200是一种免疫球蛋白超家族膜糖蛋白[17]。研究证实，CD200在CLL和MCL中具有重要的鉴别诊断价值，CLL通常高表达CD200，而MCL中CD200低表达或不表达[7],[18]。近年来，也有研究证明CD200在HCL中高表达，在滤泡性淋巴瘤、HCL-v及边缘区淋巴瘤中低表达，提示CD200在CD5^−^成熟小B细胞淋巴瘤也具有重要的鉴别诊断价值，但在国内尚无相关报道[19]–[20]。本研究聚焦于CD200在HCL和HCL-v中的表达特点，发现CD200在所有HCL中均高表达，且MFI明显增高，而在HCL-v中均不表达，与国外相关研究结果一致。本研究中5例患者免疫表型不典型，我们通过对CD200的表达特点及Annexin A1、BRAF V600E基因突变结果进行比较分析后，证明CD200可在免疫表型不典型的情况下有效区分HCL与HCL-v。HCL的分子机制是BRAF和下游MAPK/ERK信号传导途径的激活导致细胞转化、增殖并抑制细胞凋亡，该基因突变是HCL的特异性分子标志，但临床实际工作中仍有近20％的患者无法检测到BRAF V600E基因突变[21]。针对这一现象，近期有研究认为，这部分患者产生了PIK3CA和PDGFRA突变，进而导致PI3K/Akt途径被激活[22]。在本研究中，72.7％的HCL患者检测到BRAF V600E基因突变，与文献报道一致[21]。Annexin A1是一种在炎症反应中发挥复杂作用的蛋白，在HCL中高表达，但在HCL-v中几乎不表达，因此Annexin A1具有较高的灵敏度和特异性，是HCL重要的诊断标志[23]–[26]。本研究中，Annexin A1在HCL-v中均为阴性，与文献报道一致[23]–[26]，Annexin A1在HCL中的阳性表达率为81.8％，低于文献报道[23]–[26]。临床迫切需要采用新的标志物或诊断方法对此类患者进行精准诊断。MFC免疫表型分析具有高度敏感性，且简单易行，便于推广，因此在临床机构中被广泛应用。在本研究中，对于未检测到BRAF V600E基因突变和（或）Annexin A1阴性的HCL患者，我们利用MFC结合CD200、CD123和CD25的表达特点均能将HCL和HCL-v进行有效区分。

综上所述，CD200可以作为HCL与HCL-v重要的诊断标志物，可用于免疫表型不典型、未检测到BRAF V600E基因突变或Annexin A1阴性的HCL与HCL-v的鉴别诊断。由于HCL和HCL-v均为罕见的淋巴瘤，本组样本量有限，我们也将继续扩大样本量进一步研究。
